# Electrophysiological evidence of inhibited orthographic regularity effect on the recognition of real Chinese characters

**DOI:** 10.1038/srep23275

**Published:** 2016-03-23

**Authors:** Weiqi He, Cong Fan, Jie Ren, Tiantian Liu, Mingming Zhang, Wenbo Luo

**Affiliations:** 1 Research Center of Brain and Cognitive Neuroscience, Liaoning Normal University, Dalian 116029, China; 2 Laboratory of Cognition and Mental Health, Chongqing University of Arts and Sciences, Chongqing 402160, China; 3 Department of Education, Minnan Normal University, Zhangzhou 363000, China

## Abstract

Orthographic regularity is important for processing Chinese characters. However, the issues how orthographic regularity influences the visual recognition of real Chinese characters and whether common processes related to the potential effect exist between successive (SUCC) and concurrent (CONC) conditions with asynchronous presentation of S1 and S2 are still unclear. In the current study, event-related potential (ERP) technique was adopted to investigate electrophysiological correlates of the orthographic regularity effect. Behaviorally, we found fewer errors and shorter response times for SUCC and CONC conditions compared to simultaneous (SIM) condition with synchronous presentation and disappearance of S1 and S2, which demonstrates similarities between SUCC and CONC and their differences from SIM. We found bilaterally smaller N170 responses for real Chinese characters preceded by false characters compared to real characters, demonstrating that orthographic regularity may inhibit the recognition of real Chinese characters. Additionally, the inhibition effect was present in SUCC and CONC rather than SIM, which shows that smaller N170 responses may have been due to asynchronous presentations of S1 and S2 and common inhibition processes in the SUCC and CONC conditions.

The English language relies more on mappings between orthography and phonology to access meaning, whereas Chinese lacks transparency in its mapping to phonology. Orthographic information, such as orthographic regularity that heavily relies on the positional regularity of lexical radicals^1^, is critical for mastering character structures^2^. When learning Chinese characters, students who fail to grasp the positional regularity of the lexical radical may create false Chinese characters. Therefore, orthographic regularity is important for correctly recognizing Chinese characters.

What kind of impact does orthographic regularity have on real Chinese character processing? Neural adaptation refers to a decrement in neural responses to a repeated stimulus category^3^,^4^. For Chinese characters, some researchers have reported an adaptation effect as reflected by a smaller N170 component for the within-category condition than the between-category condition (alternative presentation of Chinese characters and faces)^5^. All radicals of a real Chinese character that conform to orthographic regularity are placed at legal positions, whereas radicals in false characters are all placed at illegitimate positions^1^,^2^,^6^,^7^. Additionally, a previous event-related potential (ERP) study found that real Chinese characters and pseudo-characters only elicited larger N170 responses than false characters in the left hemisphere, suggesting that real and false characters seem to be processed as different categories in the left posterior area^6^. Therefore, the orthographic (position) regularity of Chinese characters may be one of the potential factors that form different stimulus categories and produce an adaptation effect. We hypothesized that N170 changes may reflect the potential adaptation effect of orthographic regularity in real Chinese character processing.

However, there may be other ways that orthographic regularity affects the recognition of real Chinese characters. Using a picture-word paradigm, Zhang *et al*. (2009) found that compared with distractors that have different orthographies from the targets, those with orthographies similar to those of the targets facilitated performance in a Chinese naming task. This suggests that orthographic similarity facilitates picture naming in Chinese^8^. Furthermore, some researchers found priming effects related to the position information of radicals of Chinese characters^9^,^10^. Ding *et al*. (2004) reported that the prime Chinese characters only facilitated target (low-frequency complex Chinese character) recognition when the same radicals of the primes and targets were in the same positions rather than different ones^9^. Additionally, several researchers adopted ERP technique, which has a high temporal resolution, to investigate the time course of the aforementioned priming effect^10^. Su *et al*. (2012) recently used a masked priming paradigm and ERP technique to identify a priming effect due to graphical similarity and its corresponding time course. This effect was reflected by smaller P1 and N400 for target characters when positions of their radicals were the same as those of primes. Moreover, they used radicals with subordinate and dominant positions to find a priming effect of radical position preference as reflected by stronger N170 and P200 for targets with radicals in their subordinate (non-preferred) positions^10^.

Although prior studies have reported the facilitation effect of orthographic similarity^8^, the priming effect when the same radicals of the prime and target were in the same positions^9^,^10^ and the priming effect of radical position preference^10^, the issue of whether orthographic regularity (position regularity) influences the visual recognition of real Chinese characters remains unclear. As position information is important for activating radical (sublexical) information during Chinese character recognition^11^, false and real characters with illegal and legal radical positions, respectively, may have different impacts on the processing of real characters. Specifically, because radical positions of real Chinese characters are legal whereas radical positions of false Chinese characters are illegal, real characters may facilitate the processing of other real characters whereas false characters may inhibit the processing of real characters. Furthermore, the neural mechanisms of false and real character processing can be differentiated based on N170 changes^6^. Therefore, we hypothesized that N170 changes may reflect the potential facilitated orthographic regularity effect in real Chinese character processing.

For faces, several researchers used adaptation paradigm to find adaptation effect^5^,^12^,^13^,^14^,^15^ and some other researchers adopted competition paradigm to find competition effect^16^,^17^. Both of these two effects are reflected by a reduction of N170 response to faces preceded by faces rather than non-face stimuli. In addition, Kovács *et al*. (2013) compared the face-related adaptation effect with competition effect by using the successive (SUCC, in which S1 preceded S2 and disappeared when S2 appeared, similar to the adaptation paradigm of Kovács *et al*. (2006)), concurrent (CONC, in which S1 preceded S2 and they disappeared concurrently, similar to the competition paradigm of Jacques and Rossion (2007)) and simultaneous (SIM, in which S1 and S2 were synchronously presented and disappeared) conditions. They reported reduced N170 responses for subsequently presented faces (S2) when the first stimulus (S1) was a face but not a phase-scrambled face. This effect was present in both SUCC and CONC conditions with asynchronous presentation of S1 and S2, but not in SIM condition with synchronous presentation and disappearance of S1 and S2. These results show that the face N170 reduction effect results from asynchronous presentation of S1 and S2 and from common adaptation processes of successive and concurrent conditions, and that face-related adaptation and competition effects may involve the same neural mechanisms^18^. Thus, in the current study, we used SUCC, CONC and SIM conditions to explore the above potential adaptation effect of orthographic regularity of Chinese characters. If an adaptation effect of orthographic regularity exists in real Chinese character processing, we expect that common adaptation processes may be found between SUCC and CONC conditions. Moreover, in previous studies related to facilitation effects of Chinese characters, the distractor and target characters^8^ were asynchronously presented, which is similar to the SUCC and CONC conditions. Therefore, we could also employ SUCC, CONC and SIM conditions to investigate the above potential facilitation effect of orthographic regularity of Chinese characters. Thus, if a facilitation effect of orthographic regularity exists in real Chinese character processing, we would expect common facilitated processes for SUCC and CONC conditions.

In the present study, we firstly used real Chinese characters and false characters to explicitly investigate the effect of orthographic regularity in real Chinese character processing and firstly employed SUCC, CONC and SIM conditions to explore whether common processes related to the potential effect exist between SUCC and CONC conditions. To determine the time course of the potential effects, we assessed high temporal resolution ERP to record the responses induced by both real and false characters and the subsequently presented real Chinese characters. Our finding may replicate the earlier finding of greater left hemisphere responses for real Chinese characters than for false characters. In addition, if there is an effect of orthographic regularity on the processing of other real Chinese characters in both SUCC and CONC conditions, then similar N170 changes for real Chinese characters preceded by real Chinese characters compared to false Chinese characters between SUCC and CONC conditions would be observed. Behaviorally, we expect similar results between the SUCC and CONC conditions that would be different from those in the SIM condition.

## Methods

### Participants

Sixteen healthy right-handed college students (8 males, 8 females) from Chongqing, China participated in the experiment as paid volunteers. Their ages ranged from 18–23 years (mean age =20.5 years). All participants were native Chinese speakers and had an over 10-year-old experience reading Chinese characters. They were free of neurological disorders, and had normal or corrected-to-normal vision. The study was approved by Human Research Institutional Review Board at Chongqing University of Arts and Sciences, methods were carried out in accordance with its relevant guidelines and written informed consent was collected from the participants.

### Stimuli

**Figure 1:**
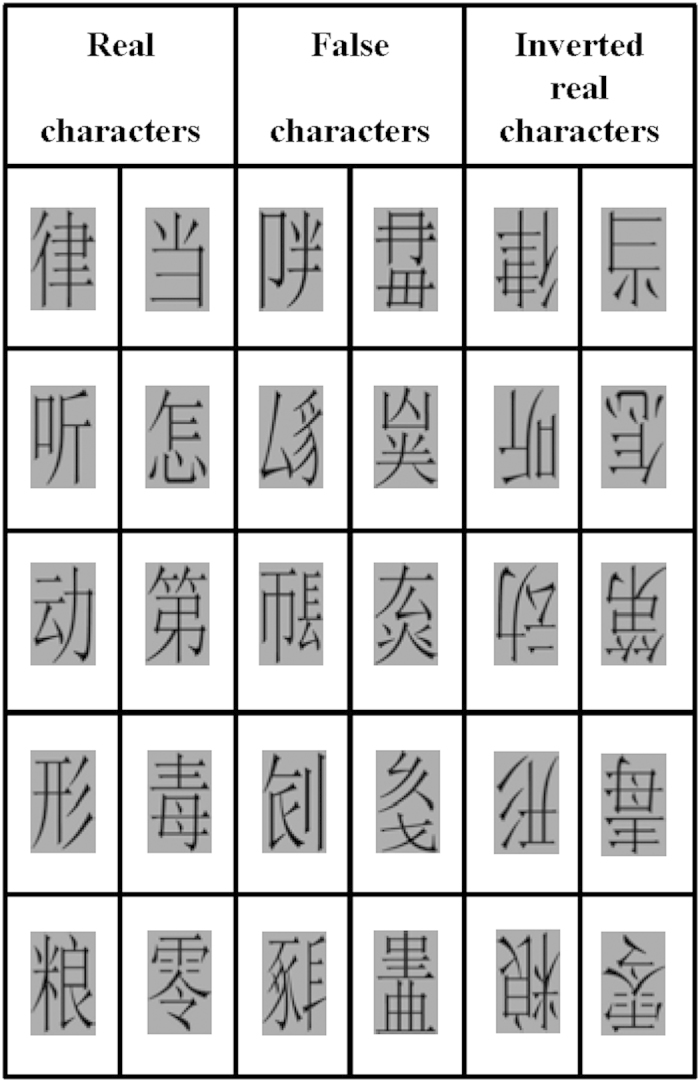
Examples of characters used in the present study.

Stimuli consisted of 50 real Chinese characters and 50 false characters (Fig. 1). Real characters were selected from the modern Chinese frequency dictionary (1985)^19^. False characters were built with existing radicals of real ones, but positions of all radicals were not legitimate. Mean stroke numbers of left-right structured real characters, up-down structured real characters, left-right structured false characters and up-down structured false characters were matched (average number =9.0; ranging from 6–13). The numbers of left-right and up-down structured characters were equivalent in both real and false characters. The average frequency of real characters was 0.58/1,000 (higher than 0.11/1,000 for each character). There was no repetition of the radicals and pronunciations among real characters and there was no repetition of the radicals among false characters. All black characters with a gray background were presented on the left or right visual field, and their closest point was at 5° from the central fixation point (when viewed 98 cm from a 17-inch screen). The resolution of the screen was 72 pixels per inch. The viewing angle of all characters was 2.8 × 3.7°. The character images were equal to one another in the luminance and contrast grade by matching to the one of the character pictures. Inverted real character pictures were created by rotating 180° of the real character pictures. Stimuli were the same in CONC, SUCC and SIM conditions.

### Procedure

**Figure 2:**
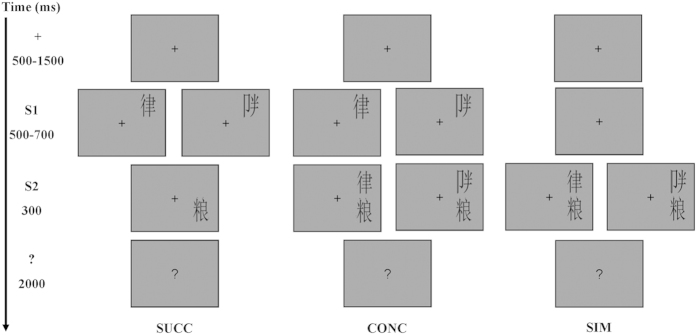
The three paradigms in the present experiment. S1 could be a real character or a false character and S2 was always a real character. SUCC: S2 appeared when S1 disappeared. S1 and S2 were always on the same side with a distance 2°. CONC: The onset of S1 was consistent with that in the SUCC condition but it remained on the screen along with S2. SIM: Onset and offset of S1 and S2 were simultaneous.

Participants were seated in a sound-isolated, dimly lit room 98 cm from a monitor. They were told to focus on the center of the screen throughout the experiment. Each trial of SUCC or CONC started with a fixation cross projected centrally for an interval ranging from 500–1500 ms. Then, a real or false character appeared either in the upper left or right visual field for a random time period of 500–700 ms. After that, a real character was presented below the first stimulus (the distance between the closest points of the two characters was 2°) for 300 ms. At the beginning of each trial of the SIM, the fixation cross appeared centrally for a random interval of 1000–2200 ms. Then, it was followed by the simultaneous presentation of the first stimulus (a real or false character) and the second stimulus (a real character) for 300 ms with same positions as those in SUCC and CONC. All three blocks (SUCC, CONC or SIM) ended with a question mark presented centrally for 2000 ms (Fig. 2), offering the participants time to accomplish a detection task. That is, inverted real characters appeared randomly in 12% of all trials as the first or second stimulus, and participants were requested to view two stimuli carefully, and to press “0” on the number keypad as fast and as accurate as possible when they appeared on the screen (Fig. 2). As inverted compound Chinese characters induced longer and larger responses than upright ones^20^, and inverted characters were used to form the task irrelevant to the hypotheses, the trials where inverted characters appeared were eliminated from the EEG analyses. In each of three blocks (SUCC, CONC, SIM), participants completed 400 trials. All characters were presented in a random order and they appeared on the left or right visual field randomly.

### Electroencephalogram (EEG) Recording

Brain electrical activity was recorded from 64 scalp electrodes on an elastic cap (Brain Products), with the left mastoid being the online reference. The activity of Vertical electrooculographic (VEOG) was collected from electrodes placed above and below the right eye, and the activity of horizontal EOG (HEOG) was recorded via electrodes fixed on the outer canthi of both eyes. All impedance was kept below 5 kΩ. Electrical activity was amplified using a 0.01–100 Hz band-pass and continuously sampled at 500 Hz/channel.

### Data Analysis

When analyzing offline with Analyzer 2.0 software (Brain Products), EEGs were rereferenced to the average of all electrode sites. Then, EEG data were filtered through a 0.1–30-Hz bandpass, segmented from −100–400 ms, and baseline-corrected with the 100 ms pre-stimulus duration. Few eye movements induced by lateralized characters were recorded throughout the experiment, and these trials were excluded from analysis. Artifacts were excluded when the amplitude of any electrode exceeded ±50 μV, and the remaining trails (more than 40 trails per condition) were used for averaging EEGs.

The P1 component is a positive-going component with a waveform peaking at around 100 ms, and the N170 component is a negative-going component with a waveform peaking at around 170 ms. As the topographical distributions of P1 and N170 components are maximal at two electrode pairs – P7/P8 located in the temporal region and PO7/PO8 located in the occipitotemporal region, and these two sets of electrodes have been used for P1^21^,^22^ and N170^23^,^24^ measurement in prior studies, we selected them for statistical analysis. After global averaging, we obtained the mean amplitudes of the P1s and N170s for each condition by using 40-ms temporal windows that were near related mean latencies. Repeated measures analysis of variance (ANOVA) was conducted on behavioral results, as well as mean amplitudes of P1 and N170 components. Statistic analysis of behavioral data (mean accuracy and reaction time) was conducted with a within-subjects factor “paradigm” (SUCC, CONC vs. SIM). When analyzing on mean amplitudes of P1 and N170 induced by the first stimulus, the factors “paradigm” (SUCC vs. CONC), “hemisphere” (left vs. right), “electrode” (P7/P8 vs. PO7/PO8), “visual field” (left vs. right), and “type of the first stimulus” (real character vs. false character) should be considered. Five-way repeated-measures ANOVAs were performed on mean amplitudes of P1 and N170 induced by the second stimulus to investigate the effect of “paradigm” (SUCC, CONC vs. SIM), “hemisphere” (left vs. right), “electrode” (P7/P8 vs. PO7/PO8), “visual field” (left vs. right), and “type of the first stimulus” (real character vs. false character). *P* values were corrected for deviations according to Greenhouse-Geisser correction. The methods were carried out in accordance with the approved guidelines.

## Results

### Behavioral results

We observed significant main effects of paradigm in terms of accuracy and response time (*F*_2,30_ = 14.36, *p* = 0, η2 p = 0.49; *F*_2,30_ = 55.48, *p* = 0, η2 p = 0.79). With regard to accuracy, pairwise comparisons revealed that there were more errors for SIM (91%) when compared to SUCC (96%) and CONC (98%). Additionally, the pairwise comparison analysis on response time demonstrated that participants performed the task faster for SUCC (452.58 ms) and CONC (440.002 ms) when compared to SIM (631.07 ms).

### ERP results

#### P1

**Figure 3:**
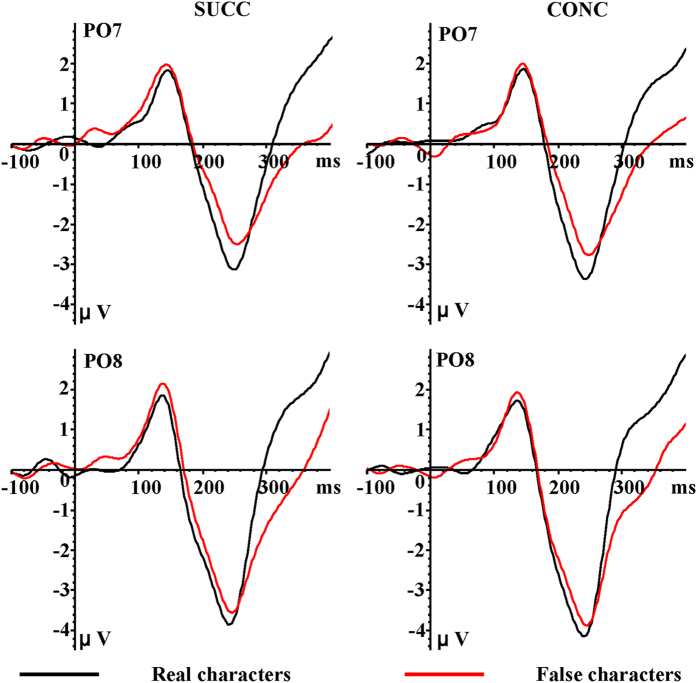
Grand average ERP waveforms for real characters (black lines) and false characters (red lines) recorded at PO7 and PO8 electrode sites.

With regard to the first stimulus (Fig. 3 and Table 1), P1 amplitudes at PO7/PO8 (1.89 μV) were larger than those at P7/P8 (1.44 μV) (*F*_1,15_ = 8.06, *p* = .01, η2 p = 0.35). There were no other significant effects, so that paradigm, hemisphere, visual field and the type of the first stimulus may not have effects on the P1 amplitude. The lack of effect between real and false characters on the P1 amplitude demonstrates that visual properties of them were successfully balanced.

**Figure 4:**
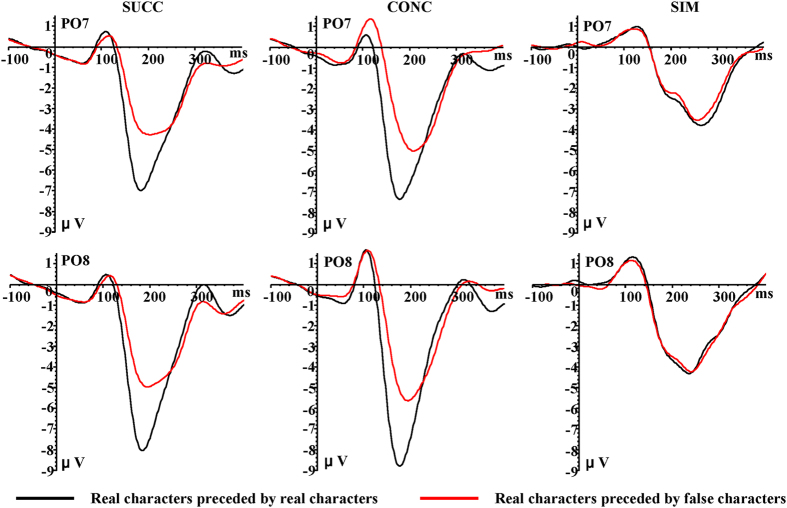
Grand average ERP waveforms for real characters preceded by real characters (black lines) and preceded by false characters (red lines) recorded at PO7 and PO8 electrode sites.

With regard to the second stimulus (Fig. 4 and Table 2), we observed larger P1 amplitudes for SIM (1.85 μV) when compared to CONC (0.75 μV), and for CONC (0.75 μV) when compared to SUCC (0.25 μV) (*F*_2,30_ = 14.05, *p* = .001, η2 p = 0.48). No other significant effects were observed, so that the type of the first stimulus may not affect the P1 amplitude induced by the second stimulus.

#### N170

When participants were viewing the first stimulus (Fig. 3 and Table 3), we observed a significant interaction between hemisphere and S1 type on the N170 amplitude (*F*_1,15_ = 4.26, *p* = 0.05, η2 p = 0.22). Simple-effects analyses revealed that real characters (−3.22 μV) evoked stronger N170 amplitudes in the left hemisphere than false characters (−2.76 μV; *p* = 0.05), and real characters (−3.80 μV) and false characters (−3.78 μV) did not have different influences on N170 amplitude in the right hemisphere (*p* = 0.91). Additionally, a significant main effect of electrode was observed (*F*_1,15_ = 8.64, *p* = 0.01, η2 p = 0.37). The pairwise comparison indicated that N170 was stronger at P7/P8 (−3.69 μV) than at PO7/PO8 (−3.09 μV).

In terms of the second stimulus (Fig. 4 and Table 4), there was a significant paradigm ×S1 type interaction on the N170 amplitude (*F*_2,30_ = 6.47, *p* = 0.01, η2 p = 0.48), with real characters eliciting stronger N170 when compared to false characters in both SUCC (real characters, −6.04 μV; false characters, −4.03 μV; *p* = 0.003) and CONC (real characters, −5.92 μV; false characters, −4.27 μV; *p* = 0.001). The SIM condition did not influence the N170 amplitude (real characters, −3.64 μV; false characters, −3.49 μV; *p* = 0.20).

## Discussion

The current study measured N170 responses to examine how orthographic regularity affects the processing of real Chinese characters and whether common processes related to the potential effect exist between SUCC and CONC. Behaviorally, we found fewer errors and shorter response times for SUCC and CONC compared to SIM, demonstrating similarities between SUCC and CONC and their differences from SIM. Our finding replicates the earlier result of greater left-lateralized N170 responses for real Chinese characters than for false characters, which indicates that the enhanced and left-lateralized character-related N170 is due to orthographic processing. The N170 response was bilaterally smaller for real Chinese characters preceded by false characters compared to real characters, indicating that orthographic regularity may inhibit the recognition of real Chinese characters. Additionally, the inhibition effect was present in SUCC and CONC but not SIM, which is similar to the common adaptation effects reported for faces between SUCC and CONC (Kovács *et al*., 2013). This finding suggests that common inhibition processes of orthographic regularity in Chinese characters may exist between SUCC and CONC.

### Inhibition effect of Chinese character orthographic regularity

Similar to a previous finding^6^, we only found greater neural responses to real Chinese characters in the left hemisphere. Thus, consistent with the prior study^6^, our results suggest that the enhancement and left lateralization of character-related N170 is elicited by processes relevant to orthographic property (i.e., radical position).

More importantly, we found that the N170 response for real Chinese characters was smaller when preceded by false rather than real characters. Prior studies investigating adaptation effects of faces reported reduced N170 elicited by faces in the face-adapted condition compared to the nonface-adapted condition^12^,^17^. Similar to the face-sensitive adaptation effect, some researchers described a Chinese-sensitive adaptation effect characterized by smaller N170 responses for Chinese characters in the within-category condition than the between-category condition^5^. These previous studies support the notion that neural adaptation involves reduced neural responses for a repeated stimulus category. Inconsistent with prior results, we observed stronger neural responses for real Chinese characters when the category was repeated; this may be because real and false characters are not in different categories.

Instead of an adaptation effect, we measured an inhibition effect of orthographic regularity in real Chinese character processing. Su *et al*. (2012) found a priming effect induced by radical position preference that was reflected by stronger N170 and P200 components. Notably, greater N170 and P200 for subordinate radical position characters are thought to indicate that processing these characters requires more effort^10^. Unlike the prior study, we firstly employed real and false characters and SUCC, CONC and SIM conditions to find larger N170 responses for real Chinese characters preceded by real compared to false characters. According to the explanation of the findings of the previous study^10^, our interpretation is that for skilled native speakers, when S1 is a false character rather than a real character, less attention is paid to the content of subsequently presented real characters because they are in a context that lacks semantics. As a result, the N170 is smaller for real characters preceded by a false character rather than a real character. Thus, compared to real characters, false characters may inhibit the recognition of other real characters, and N170 changes can reflect the inhibition effect. Although N170 amplitudes elicited by S1 were only different in the left hemisphere, we found a bilateral N170 effect induced by S2. This may be because S2 was always a real Chinese character whereas S1 was either a real character or a false character. The N170 component induced by S2 in SIM when S1 and S2 were presented simultaneously and the ERP response induced by S2 in SUCC and CONC could be N170 with a shorter latency because of the preceding presentation of S1. Collectively, these findings suggest that there is an inhibition effect of orthographic regularity in real Chinese characters that can be reflected by N170 changes.

### Similarities between SUCC and CONC and their differences from SIM

With regard to the second stimulus, we found larger P1 amplitudes for SIM than for CONC, which was greater than for SUCC. This may be because everything is new in the SIM condition without priming when compared to CONC and SUCC conditions, and CONC has more physically visual items than SUCC. More attentional resources are recruited for new or additional “stuff,” leading to larger P1 amplitudes.

Interestingly, our behavioral findings of fewer errors and shorter response times for SUCC and CONC compared to SIM may indicate that SUCC is similar to CONC and that both SUCC and CONC are different from SIM. Furthermore, we only observed a smaller N170 for real Chinese characters preceded by false characters rather than real characters in the SUCC and CONC conditions, and this reduction was not different between the two conditions. Findings using an adaptation paradigm^13^ similar to SUCC were similar to the results of a study that employed a competition paradigm^17^ similar to the CONC. Furthermore, a previous study reported similar N170 changes for faces in the SUCC and CONC conditions^18^. Similar to the prior study^18^, we found similar N170 responses in the SUCC and CONC conditions. S1 disappeared when S2 started to appear in SUCC, whereas S1 remained on the screen when S2 started to appear. Nevertheless, S2 was preceded by S1 in both the SUCC and CONC conditions. Thus, the similar results in the SUCC and CONC conditions indicate that the finding is dependent on asynchronous presentations of S1 and S2 and common processes in the SUCC and CONC conditions; whether S1 disappears at the onset of S2 does not have an effect. Our finding of similar N170 responses for S2 in the real and false character conditions of SIM is in accordance with a previous study that found that similar and dissimilar face pairs elicited similar ERPs until 250 ms after their onsets in a paradigm similar to SIM^25^. This may be because S1 and S2 are presented concurrently in the SIM condition, which is different from the SUCC and CONC conditions with asynchronous S1 and S2 presentations. Thus, in the current study, similar N170 responses in SUCC and CONC rather than SIM may have been due to asynchronous S1 and S2 presentations and common inhibition processes in the SUCC and CONC conditions.

Our study had several limitations. Firstly, other investigations of the adaptation effects of faces reported reduced N170 responses elicited by faces in the face-adapted condition compared to the phase-randomized face^13^,^26^ or nonface-adapted condition^5^,^12^. Stroke combinations or noncharacter objects can be added in future studies to determine whether an adaptation effect exists in Chinese character processing. Secondly, although the possibility of radical consistency of S1 and S2 is small (0.96% per event), the same radicals of S1 and S2 may influence the inhibition effect of orthographic regularity in Chinese characters. Thus, the factor of radical consistency should also be assessed. Thirdly, as preschoolers and skilled readers have different abilities to grasp the orthographic regularity of Chinese characters, it would be worthwhile to investigate the effect of orthographic regularity during Chinese character learning.

## Conclusion

In the current study, we examined how orthographic regularity influences the processing of real Chinese characters and whether common processes related to the potential effect exist between SUCC and CONC conditions. Our behavioral finding of fewer errors and shorter response times for SUCC and CONC compared to SIM shows similarities between SUCC and CONC and their differences from SIM. Our findings replicate the earlier finding of greater neural responses for real Chinese characters than for false characters in the left hemisphere, which indicates that the enhancement and left lateralization of character-related N170 is elicited by processes relevant to orthographic property (i.e., radical position). Additionally, we found that orthographic regularity may inhibit the recognition of real Chinese characters, which is reflected by bilaterally smaller N170 responses for real Chinese characters preceded by false characters relative to real characters. In addition, the inhibition effect was present in the SUCC and CONC conditions rather than SIM, showing that smaller N170 responses may have been due to asynchronous presentations of S1 and S2 and common inhibition processes in the SUCC and CONC conditions.

## Additional Information

**How to cite this article**: He, W. *et al*. Electrophysiological evidence of inhibited orthographic regularity effect on the recognition of real Chinese characters. *Sci. Rep*. **6**, 23275; doi: 10.1038/srep23275 (2016).

## Figures and Tables

**Table 1:** Mean amplitudes (μV) (±SD) of P1 induced by stimulus 1 for each condition.

	Left visual field								Right visual field							
	Left hemisphere				Right hemisphere				Left hemisphere				Right hemisphere			
	real character		false character		real character		false character		real character		false character		real character		false character	
	P7	PO7	P7	PO7	P8	PO8	P8	PO8	P7	PO7	P7	PO7	P8	PO8	P8	PO8
SUCC	1.94	2.38	2.05	2.64	1.03	1.27	1.21	1.56	0.79	1.06	1.00	1.36	1.70	2.32	1.96	2.63
	(1.39)	(1.90)	(1.41)	(1.83)	(1.33)	(1.66)	(1.47)	(1.88)	(1.13)	(1.48)	(0.97)	(1.29)	(1.48)	(1.87)	(1.21)	(1.64)
CONC	1.95	2.42	2.09	2.36	1.28	1.46	0.95	1.37	0.53	0.87	0.81	1.29	1.90	2.58	1.90	2.67
	(1.15)	(1.56)	(1.44)	(1.93)	(1.24)	(1.72)	(1.44)	(1.99)	(1.79)	(2.06)	(1.43)	(1.51)	(1.44)	(2.00)	(1.43)	(1.94)

**Table 2:** Mean amplitudes (μV) (±SD) of P1 induced by stimulus 2 for each condition.

	Left visual field								Right visual field							
	Left hemisphere				Right hemisphere				Left hemisphere				Right hemisphere			
	real character		false character		real character		false character		real character		false character		real character		false character	
	P7	PO7	P7	PO7	P8	PO8	P8	PO8	P7	PO7	P7	PO7	P8	PO8	P8	PO8
SUCC	−0.17	−0.23	0.03	−0.01	0.75	1.25	0.30	0.65	−0.06	0.39	0.18	0.39	0.30	0.19	0.09	−0.02
	(1.64)	(2.29)	(1.22)	(1.64)	(1.66)	(1.88)	(1.41)	(1.72)	(1.30)	(1.56)	(1.34)	(1.46)	(1.74)	(2.01)	(1.71)	(2.07)
CONC	0.05	−0.00	0.83	1.18	1.20	1.79	1.10	1.96	0.08	0.40	0.72	1.14	0.30	0.03	0.46	0.68
	(1.53)	(2.28)	(1.42)	(1.96)	(1.62)	(1.86)	(1.62)	(1.82)	(1.32)	(1.82)	(1.28)	(1.48)	(1.91)	(2.54)	(1.87)	(2.05)
SIM	2.39	2.70	2.33	2.64	1.39	1.56	1.37	1.74	0.72	1.18	0.61	0.94	2.48	2.81	2.22	2.47
	(1.70)	(1.77)	(1.17)	(1.41)	(0.99)	(1.06)	(1.26)	(1.32)	(1.31)	(1.42)	(1.36)	(1.44)	(1.57)	(2.24)	(1.34)	(1.95)

**Table 3:** Mean amplitudes (μV) (±SD) of N170 induced by stimulus 1 for each condition.

	Left visual field								Right visual field							
	Left hemisphere				Right hemisphere				Left hemisphere				Right hemisphere			
	real character		false character		real character		false character		real character		false character		real character		false character	
	P7	PO7	P7	PO7	P8	PO8	P8	PO8	P7	PO7	P7	PO7	P8	PO8	P8	PO8
SUCC	−3.50	−2.98	−3.09	−2.36	−3.84	−2.87	−3.93	−2.80	−3.32	−2.88	−2.98	−2.39	−4.53	−3.98	−4.43	−3.91
	(2.35)	(2.30)	(2.48)	(2.39)	(3.36)	(3.36)	(3.65)	(3.58)	(2.28)	(2.60)	(2.88)	(3.02)	(2.92)	(3.06)	(3.25)	(3.01)
CONC	−3.58	−3.10	−3.29	−2.86	−3.74	−3.18	−4.22	−3.25	−3.35	−3.05	−2.92	−2.23	−4.26	−4.03	−4.11	−3.56
	(1.95)	(1.66)	(2.63)	(2.44)	(3.14)	(3.18)	(3.96)	(4.01)	(2.48)	(2.85)	(2.93)	(3.42)	(3.67)	(3.95)	(3.85)	(3.77)

**Table 4:** Mean amplitudes (μV) (±SD) of N170 induced by stimulus 2 for each condition.

	Left visual field								Right visual field							
	Left hemisphere				Right hemisphere				Left hemisphere				Right hemisphere			
	real character		false character		real character		false character		real character		false character		real character		false character	
	P7	PO7	P7	PO7	P8	PO8	P8	PO8	P7	PO7	P7	PO7	P8	PO8	P8	PO8
SUCC	−4.71	−5.57	−3.20	−3.78	−6.68	−7.06	−4.63	−4.78	−5.67	−6.40	−3.30	−3.89	−5.80	−6.44	−4.23	−4.45
	(3.15)	(3.69)	(2.18)	(2.51)	(3.38)	(3.62)	(2.25)	(2.49)	(3.22)	(3.91)	(2.31)	(2.63)	(2.90)	(3.11)	(2.24)	(2.34)
CONC	−4.83	−5.58	−3.48	−3.87	−6.08	−6.50	−4.86	−4.78	−5.67	−6.27	−3.98	−4.29	−5.89	−6.51	−4.28	−4.57
	(2.69)	(3.24)	(1.59)	(2.21)	(2.41)	(2.36)	(2.13)	(1.92)	(2.93)	(3.40)	(1.52)	(2.18)	(2.13)	(2.99)	(2.39)	(2.39)
SIM	−3.93	−3.52	−3.66	−3.37	−3.64	−2.95	−3.72	−3.11	−3.59	−2.99	−3.15	−2.54	−4.27	−4.21	−4.22	−409
	(2.14)	(2.13)	(2.13)	(2.09)	(3.25)	(3.74)	(3.11)	(3.63)	(2.34)	(2.74)	(2.37)	(2.55)	(2.79)	(3.24)	(3.22)	(3.36)
